# Bidirectional Relationship between Gastric Emptying and Plasma Glucose Control in Normoglycemic Individuals and Diabetic Patients

**DOI:** 10.1155/2018/1736959

**Published:** 2018-10-03

**Authors:** Bogdan Mircea Mihai, Cătălina Mihai, Cristina Cijevschi-Prelipcean, Elena-Daniela Grigorescu, Mihaela Dranga, Vasile Drug, Ioan Sporea, Cristina Mihaela Lăcătușu

**Affiliations:** ^1^“Grigore T. Popa” University of Medicine and Pharmacy, Clinical Centre of Diabetes, Nutrition and Metabolic Diseases, “Sf. Spiridon” Clinical Hospital, Iași, Romania; ^2^“Grigore T. Popa” University of Medicine and Pharmacy, Institute of Gastroenterology and Hepatology, “Sf. Spiridon” Clinical Hospital, Iași, Romania; ^3^Gastroenterology, “Victor Babes” University of Medicine and Pharmacy Timișoara, Romania

## Abstract

Gastric emptying and glycemic control pathways are closely interrelated processes. Gastric chyme is transferred into the duodenum with velocities depending on its solid or liquid state, as well as on its caloric and nutritional composition. Once nutrients enter the intestine, the secretion of incretins (hormonal products of intestinal cells) is stimulated. Among incretins, glucagon-like peptide-1 (GLP-1) has multiple glycemic-regulatory effects that include delayed gastric emptying, thus triggering a feedback loop lowering postprandial serum glucose levels. Glycemic values also influence gastric emptying; hyperglycemia slows it down, and hypoglycemia accelerates it, both limiting glycemic fluctuations. Disordered gastric emptying in diabetes mellitus is understood today as a complex pathophysiological condition, with both irreversible and reversible components and high intra- and interindividual variability of time span and clinical features. While limited delays may be useful for reducing postprandial hyperglycemias, severely hindered gastric emptying may be associated with higher glycemic variability and worsened long-term glycemic control. Therapeutic approaches for both gastric emptying and glycemic control include dietary modifications of meal structure or content and drugs acting as GLP-1 receptor agonists. In the foreseeable future, we will probably witness a wider range of dietary interventions and more incretin-based medications used for restoring both gastric emptying and glycemic levels to nearly physiological levels.

## 1. Introduction

Gastric emptying and glycemic variations are interdependent processes influenced by the incretin system, the solid or liquid state of ingested nourishment, and the macronutrient composition of food [1–3]. In diabetic patients, gastric emptying may be either accelerated or delayed, with irregular intra- and interindividual fluctuations in the rate and clinical expression [4, 5]. Gastric emptying also interacts with overall and postprandial glycemic control [6, 7]. Therapeutic approaches aiming to correct both gastric emptying and variations of serum glucose levels include diet changes in meal sequence or content and incretin-based medications [8–11].

The relation between the stomach and diabetes mellitus was alleged ever since ancient times. In the first century AD, Aretaeus of Cappadocia, whose reputation is due to his work related to diabetes more than any other physician in antiquity, would say “We must, therefore, strengthen the stomach by all means, which is the fountain of thirst” when speaking of diabetes mellitus treatment [12]. This hypothesis is obviously no longer valid in the 21st century, but the close bond between gastric emptying and glycemic control is recognized today as a reality beyond any doubt. This relationship is bidirectional; on the one hand, gastric emptying is influenced by glycemic control (as hyperglycemic values slow it down and hypoglycemia accelerates it), while on the other hand, gastric emptying may influence glycemic values, particularly postprandial ones [13, 14].

This review focuses on the current knowledge of the influences gastric emptying and glycemic fluctuations exert upon each other. The paper outlines the physiology and assessment of gastric emptying, diabetes-induced disorders of this relationship, but also the influence of nutrients and GLP-1 receptor agonists on gastric emptying and glycemic control.

## 2. Physiologic Control of Gastric Emptying

When foods enter the stomach, the proximal gastric region initially relaxes in order to “accommodate” the ingested nourishments. They subsequently reach the distal areas of the stomach and, by means of antral contractions, are ground and mixed with the gastric hydrochloric acid secretion. Gastric chyme (Greek: *khymos* = juice), a semifluid mass, is thus formed. When the resulting particles are less than 1–2 mm in diameter, they go through the pylorus into the duodenum. Their transit is backed by antral contractions and relaxation of the pyloric sphincter. In normal conditions, the rate of gastric emptying may vary between 1 to 4 kcal/min [1], depending on the composition of gastric content (solids or liquids) and the macronutrient type [2]. As a result of their high caloric content, lipids are evacuated more slowly from the stomach than carbohydrates or proteins [15]. Digestion of solids begins after feeding with a lag time of 20 to 40 minutes, required for their grounding into 1–2 mm diameter particles, and then, a quasilinear gastric emptying begins. Therefore, their evacuation from the stomach begins approximately 40 minutes after food intake and may last for a few hours. Evacuation of liquids is immediate (without any lag phase) and usually monoexponential. The more nutritionally dense the liquids are, the slower the gastric emptying becomes [3]. In fact, caloric content of foods may exert a greater influence on gastric emptying than previously thought. Some recent results suggest not only liquids of equal energetic densities are evacuated from the stomach with similar speeds, but meals with the same caloric content given with equal amounts of water have nearly identical gastric emptying curves, no matter if their initial form was solid or liquid [16–18].

Nearly thirty years ago, Malbert and Ruckebusch described an intermittent transpyloric flux, with antral contractions and pyloric relaxation turning up at approximately 20-second intervals, while duodenal flux was continuous and uniform [19]. At that moment, the mechanical function of the duodenal bulb was thought to be the only factor to transform this flux from intermittent to continuous. The mechanism is in fact far more complex. When nutrients reach the intestine, they become a signal stimulating the blood release of intestinal hormones known as the incretin system. K cells from the upper intestine (duodenum) secrete *glucose-dependent insulinotropic peptide* (GIP); in the distal segment of the intestine, L cells secrete *glucagon-like peptide-1* (GLP-1). Both GLP-1 and GIP have glucose-dependent insulin secretion effects; GLP-1 inhibits glucagon secretion, and GIP exerts glucagonotropic actions [3, 20–22]. While it was demonstrated that GIP has no effect on gastric emptying, GLP-1 induces an inhibitory feedback effect, delaying gastric emptying [22]. Recent data also suggest a relation between glycemic values and stomach emptying [13, 23]. High velocity gastric emptying allows nutrients to reach the intestine more rapidly, thus increasing postprandial glycemia; on the other hand, hyperglycemia delays stomach emptying, so the nutrients are propelled more slowly for absorption at the intestinal level [14]. Hypoglycemia induces reverse effects, by accelerating gastric emptying and increasing the nutrient absorption speed, thus allowing for a more prompt correction of glycemic levels [1]. The ability to increase gastric emptying was found to persist in healthy individuals even after repeated hypoglycemic episodes [24]. This is in contrast to other hypoglycemia-induced reactions, such as the clinical signs induced by adrenergic response, which are subdued by impaired hormonal counterregulation and tend to fade out during recurrent hypoglycemias.

## 3. Assessment of Gastric Emptying

The first measurement of gastric emptying dates back to the Middle Ages, when Frederic II Hohenstaufen, the leader of the Holy Roman Empire, ordered two prisoners to be well fed and then sent one of them to hunt and let the other one rest. The two men were then executed, and gastric content was analysed in both cases; the man who went hunting had a full stomach, while the second man's stomach was empty [25]. Activity of the sympathetic nervous system had been predominant in the man who went hunting and had physical exercise, inducing relaxation of gastrointestinal muscularity and sphincter contraction, while the other showed a predominance of the parasympathetic nervous system, with opposite effects.

At present, multiple methods of measuring gastric emptying exist. As a general condition, temporary discontinuation of prokinetics, anticholinergics, opiates, or any other medication exerting an influence on gastric motility is needed whenever possible; in order to avoid the delaying effect of high glycemic values, some investigation protocols require the procedures to be performed at fasting glucose levels below 15.3 mmol/L (275 mg/dL) [26, 27]. Among these methods, the golden standard is scintigraphy performed after labelling solid and/or liquid food with radiotracers (technetium, indium) [28]. However, standardization of the method was only recently accomplished, with current guidelines advising for the administration of a specified low-fat meal (egg white from two large eggs, two slices of bread with jam and water) labelled with ^99m^technetium sulphur colloid and for the measurement of gastric content after 1, 2, and 4 hours. These technical conditions were fulfilled, and a gastric retention of more than 60% after 2 hours or 10% after 4 hours is considered pathological [29, 30].

Breath tests are methods seemingly as good as scintigraphy [31]. Food is labelled with ^13^carbon (^13^C); after food is evacuated from the stomach, ^13^C enters the intestine, is absorbed into bloodstream, and reaches the liver, being transformed into ^13^CO_2_. Determination of ^13^CO_2_ in exhaled air provides indirect data on gastric emptying. This method has the advantage of not involving radiations, thus being allowed in children and pregnant women [31].

2D or 3D ultrasonography is a useful method, but it requires considerable amounts of patience and experience from the examiner [32, 33].

Another modern alternative is the use of a wireless motility capsule, a 26 × 13 mm medical device with pressure, temperature, and pH-measuring sensors, approved in 2006 by the Food and Drug Administration for the study of gastric emptying when gastroparesis is suspected. A sudden increase in pH values marks the moment when the capsule exits the stomach and enters the duodenal region; values greater than 5 hours are considered representative for delayed gastric emptying. The wireless motility capsule may be used in ambulatory settings; it has the advantage of avoiding exposure to radiations, but its use is limited in dysphagia or possible bowel obstruction, due to its size [26]. Comparison between gastric emptying time measured by the wireless motility capsule and the golden standard of scintigraphy yielded good results in healthy individuals and patients with gastroparesis [34].

## 4. Causes of Gastric Emptying Disorders in Diabetes

Disordered gastric emptying in diabetic patients must be understood in terms of factors controlling gastrointestinal motility. A normal evacuation of the stomach involves coordination between contractile activities of its proximal and distal regions, pylorus, and proximal duodenum. These movements are controlled by the enteric and autonomic nervous systems, as well as by neurohormonal pathways. The enteric nervous system is represented by Cajal interstitial cells, belonging to the Auerbach nerve plexus within the muscle layer of the digestive tube. The enteric nervous system comprises over 10^8^ neurons, thus being considered an authentic brain of the digestive tract; Cajal interstitial cells operate as a pacemaker. The motor activity of the digestive tube is modulated by the autonomic (extrinsic) nervous system; the parasympathetic nervous system induces the contraction of intestinal musculature and sphincter relaxation, while the sympathetic nervous system elicits opposite actions. Among neurohormonal pathways, acetylcholine and substance P promote muscular contraction, while nitric oxide, carbon monoxide, and vasoactive intestinal polypeptide exert inhibitory actions [35, 36].

Until a few years ago, the traditional paradigm on delayed gastric emptying in diabetic patients considered it to be induced exclusively by vagal neuropathy and to be irreversible. Recent data suggest a much more complex process, influenced by multiple pathophysiologic mechanisms which are not yet completely understood; some are reversible, and some are not [32, 37]. Loss or dysfunction of Cajal interstitial cells plays a major pathophysiologic part in the induction of abnormal gastric emptying under diabetic conditions. Anomalies of enteric nerves, immune system, or nitric oxide synthase (inducing low nitric oxide levels) and low levels of carbon monoxide-producing heme oxygenase, which protects Cajal interstitial cells from oxidative stress, are also involved [3, 38]. Recent experimental data attribute damage of myenteric cholinergic neurons and interstitial cells of Cajal to impaired signalling through the axes of insulin and insulin-like growth factor-1 receptors [32, 39].

The term *gastroparesis diabeticorum* was coined by Kassander in 1958 [40]. Recent publications shed new light on our knowledge about this complication of diabetes mellitus. Delayed gastric emptying in diabetic patients may often be merely mild or moderate, and some persons may even exhibit accelerated propulsion of the stomach content into the duodenum. Considering this, the term *gastroparesis* should be limited to those severe clinical forms displaying symptoms and an important delay of gastric emptying, where food consumed many hours or days before is found in vomit or in aspiration liquid [38]. The relationship between the speed of gastric emptying and the symptoms is weak, with some patients having severe symptoms but only minor delays in stomach emptying, while others may have a severely delayed gastric emptying and no symptoms at all [41]. Moreover, it is commonly acknowledged today that more severe delays in gastric emptying or aggravations of symptoms may not appear even after many years of evolution [42].

More than 20 years ago, Horowitz et al. described a delayed gastric emptying for solids as well as liquids in patients with diabetic gastroparesis [43]. His team reported an increased prevalence of delayed gastric emptying in individuals with long-term type 1 or type 2 diabetes, despite significant fluctuations in gastric emptying from one individual to another, both in diabetic patients and in nondiabetic control subjects [4].

Recent research brought forth a change in paradigm on delayed gastric emptying. If this delay is limited, its effects may even be beneficial by the protection it offers against glycemic variations. This concept is not entirely new; glucose perfusions were reported to suppress “hunger” contractions ever since 1956 [44]. Results of scintigraphic measurements published during the 90s showed that hyperglycemia delays gastric emptying in patients with type 1 diabetes for both solids and liquids [27]. In diabetic conditions, these variations act as a buffer against serum glucose fluctuations; when glycemic values are elevated, gastric emptying is delayed in order to avoid supplementary amounts of glucose reaching the intestine in a short time, thus increasing glycemia even more. On the contrary, hypoglycemia would need more glucose entering the bloodstream as quickly as possible; therefore, a faster gastric emptying, rapidly sending glucose into the duodenum for absorption, is beneficial for the organism. Scintigraphic measurements also proved the validity of such a mechanism in various clinical settings. Glycemic values of approximately 2.8 mmol/L (50 mg/dL) increase the rate of gastric emptying in patients with type 1 diabetes [5]. Delayed gastric emptying was also described in individuals recently diagnosed with type 2 diabetes and with mean HbA_1c_ values of 10.5%; after 3 months of antihyperglycemic therapy, allowing HbA_1c_ values to be reduced at approximately 5%, a new scintigraphy was performed, showing an increase in gastric emptying nearing the evacuation speed of nondiabetic persons [45].

## 5. Glycemic Responses Induced by Fluctuations of Gastric Emptying

As mentioned before, gastric emptying establishes a bidirectional relationship with glycemic levels: glycemia influences gastric emptying, while the latter may also influence the value of postprandial glycemia. Postprandial serum levels of glucose are essential in diabetic patients. First, postprandial hyperglycemia is associated with increased oxidative stress and thus directly involved in the pathogenesis of chronic micro- and macrovascular complications of diabetes mellitus [46]. Second, data published by Monnier et al., showing postprandial hyperglycemia to be an important constituent of glycemic control, particularly in patients with only slightly increased HbA_1c_ values, are seen today as undisputed common knowledge [47]. Third, lifestyle in developed industrialized countries involves the ingestion of approximately 2500 kilocalories per day, split in 2 or 3 daily meals. Since the rate of gastric emptying is 1 to 4 kilocalories/minute, it results that modern mankind spends almost all the time in a postprandial or postabsorptive state, with only 3 or 4 hours early in the morning spent in an authentic fasting state [38]. All these considerations highlight the importance of postprandial hyperglycemia.

Several research teams found a direct relationship between the rate of gastric emptying and postprandial serum glucose levels [6, 7, 48, 49]. In patients with type 1 diabetes, altered rates of gastric emptying may impair efforts to adjust doses of prandial insulin according to the amounts of ingested nutrients. The most difficult problem in patients with type 1 diabetes is not the issue of too high or too low speeds of gastric emptying but its unpredictability. Most authors found that gastric emptying is increased even after recurrent episodes of hypoglycemia, not only in healthy individuals [24] but also in diabetic patients [50]. On the contrary, Lysy et al. evaluated gastric emptying in insulin-treated diabetic persons with frequent hypoglycemia and found it to be delayed in most situations [51]; in fact, such individuals exhibit discrepancies between the action of prandial insulin and the rates of gastric emptying [52]. Moreover, Parthasarathy et al. found delayed gastric emptying (even though associated with lesser postprandial hyperglycemia) to paradoxically induce a tendency towards higher glycemic levels throughout the day at continuous glucose monitoring measurements and therefore to favour, in time, a worsened glycemic control [53]. Hereupon, the evaluation of diabetic patients with frequent hypoglycemia should best include the assessment of gastric emptying; the “gastric hypoglycemia” (by delayed gastric emptying) proves to be an important concept in the management of diabetes mellitus [53].

## 6. Effects of GLP-1 on Gastric Emptying

As seen before, GLP-1 is an incretin hormone secreted by L cells in the distal intestine and having both the roles of increasing insulin secretion (secretagogue) and reducing glucagon secretion, as well as the effect of delaying gastric emptying [54–56]. When gastric emptying is fast, high amounts of GLP-1 are synthesized and bring it back to normal by a mechanism of inhibitory feedback. In the postprandial state, the main effect of GLP-1 is not the stimulation of insulin secretion but lowering of postprandial glycemia by means of delayed gastric emptying [57]. When intravenous erythromycin was used to counteract the delayed gastric emptying induced by a GLP-1 infusion in healthy individuals after a liquid test meal, the insulin secretory response and postprandial glucose concentrations were reversed and brought close to the levels seen during placebo administration, thus proving its main effect on gastric emptying [58].

GLP-1 effect on gastric emptying after a long-term, continuous use was proven recently to fade by a phenomenon of tachyphylaxis. On the contrary, insulinotropic and glucagonostatic actions of GLP-1 are not modified even after a long-term use. The mechanism causing the difference between the evolution of GLP-1 effects over time is not known [59, 60].

## 7. Influence of Nutrients on Gastric Emptying and Glycemic Levels

Nutritional factors are frequently overlooked, and discussion is often limited to other physiological or pharmacological issues in publications analysing the correlations between gastric emptying and glycemic values. However, effects exerted by the main nutrients or other food components on both these aspects can in fact hardly be separated from other perspectives, given that both gastric emptying and postprandial glycemic values imply the coexistence of meals and therefore nutrients. It is our team's opinion, supported by our double professional expertise in the fields of both clinical nutrition and diabetes care, that a complete description of the multilateral relationship existing between gastric emptying and glycemic levels and regulation should always include referrals to the influence exerted by food principles.

Adding sources rich in proteins to carbohydrate-based meals determines a 20% to 30% reduction in postprandial glucose levels [61]. Besides the stimulation of insulin secretion (driven by direct stimulatory effects, but also indirectly, through an increased incretin response), underlying pathways include delayed gastric emptying under the influence of the same incretin hormones [62]. Whey or soy proteins seem to give the best responses [8, 63], but favourable effects on postprandial glycemic levels were also reported for rice, pea, and oat proteins [64]. Giezenaar et al. found whey protein drinks to slow gastric emptying and alter insulin, glucagon, GLP-1, and GIP secretions in older men and women [65]. The structure of whey and soy proteins is rich in branched-chain amino acids, allowing faster digestion and absorption times, and therefore a quicker insulin release from pancreatic beta cells [66]. Ma et al. administered whey-based drinks to type 2 diabetes patients and proved a reduction of 50% or 40% in postprandial blood glucose areas under the curves if drinks were ingested 30 minutes before meals or when meal began, respectively [67]. Jakubowicz et al. found a 28% reduction in postprandial glycemic levels and increased responses in insulin, C-peptide, GLP-1, and GIP secretions when a whey preload was administered before a high glycemic index breakfast to a group of well-controlled type 2 diabetic patients [68].

In contrast with whole-structure proteins, intragastric administration of isolated amino acids such as lysine, leucine, or isoleucine does not seem to influence gastric emptying, even though it may reduce postprandial glycemic levels, most probably by direct stimulation of insulin secretion [69, 70]. However, paradoxical results are also published, leaving the debate about free amino acid effects on gastric emptying still open [71, 72]. Other three amino acids, histidine, glutamate, and aspartate, were reported to increase both postprandial glycemic levels, velocity of gastric emptying, and GLP-1 serum concentrations [71]. L-Tryptophan isomeric form was found to significantly delay gastric emptying, even though the effect on GLP-1 secretion was minimal [72].

Classical nutrition information considers that meals with high lipid content reduce the velocity of gastric emptying [15]. Recent data seem to confirm this theory by showing, for example, that high-fat meals may worsen symptoms of diabetic gastroparesis when compared to low-fat ones [73]. More than the absolute lipid load, the degree of emulsification and the lipid droplet size seem to influence gastric emptying. Tan et al. showed that fine emulsions of olive oil in water slowed gastric emptying more than a coarse emulsion or a nonemulsified mixture of olive oil and water [9].

Dietary fiber-rich foods are also able to reduce postprandial glycemia, with soluble fiber exerting the most pronounced effect. Most common explanations usually refer to an unmediated ability of soluble fiber to delay glucose absorption [74]. However, Repin et al. recently found that multiple types and doses of soluble fiber are able to induce similar reductions in postprandial glycemia and insulinemia, concluding that a modified meal viscosity and maybe a slower gastric emptying are involved [75]. Steinert et al. reported the use of oat bran mixed in water as a preload before a white bread test meal to efficiently reduce the postprandial glycemic area under curve and speculated it may be correlated to a delayed gastric emptying [76]. The comparison between effects induced by ingestion of proportional amounts of porridge based on either oat flakes or oat flour with the same protein, fat, and carbohydrate content showed lower levels of postprandial glycemia and a slower gastric emptying for oat flake porridge, explained by its conserved fiber structure [77]. However, the exact magnitude and conditionality of the effect fibers may exert on gastric emptying are still debatable. Contrary to previous evidences, a small study found that polydextrose, a low-viscosity soluble fiber, was able to reduce energy intake and postprandial glycemic and insulinemic responses but not to significantly modify gastric emptying [78].

## 8. Effects of GLP-1 Receptor Agonists on Gastric Emptying and Glycemic Levels

New knowledge on incretin hormones allowed the development of new drug therapies including GLP-1 receptor agonists. This class currently includes multiple representatives, some in clinical use and others in various stages of development [79]. GLP-1 receptor agonists are classified based on their half-life; the short-acting agents are designated as prandial agonists (exenatide BID, lixisenatide), while long-acting agents are considered nonprandial agonists (exenatide QW, liraglutide). Their glycemic effects differ, as prandial agonists mostly influence postprandial glycemia and nonprandial agonists exert a greater effect on fasting glycemia [10, 11, 80]. As nonprandial GLP-1 receptor agonists have longer half-lives and prolonged action, the gastric emptying effect is reduced by tachyphylaxis and their influence on postprandial glycemia is thus diminished by comparison with prandial agonists [81–84]. However, effects of short-acting and long-acting GLP-1 receptor agonists on postprandial glycemia are not always different, since semaglutide, a longer-duration GLP-1 receptor agonist recently approved in the United States by Food and Drug Administration, seems able to lower postprandial glycemia and the velocity of gastric emptying in obese subjects [85].

Novel therapeutic guidelines for type 2 diabetes recommend the association of prandial GLP-1 receptor agonists to basal insulin; benefits of such pharmacologic combinations bring together the predominant effect of basal insulin on fasting glycemia and the effect of prandial GLP-1 receptor agonists on postprandial glycemia, based on their ability to inhibit gastric emptying [86, 87]. This association between basal insulin and prandial GLP-1 receptor agonists is preferred today to the classical intensification of basal insulin therapy by adding prandial insulins, as it offers advantages of both a lower risk for hypoglycemia and a reduced weight gain [88, 89].

The effect of GLP-1 receptor agonists in diabetic patients with autonomic neuropathy and delayed gastric emptying, even though less studied, is usually feared to be deleterious in clinical practice by inducing or aggravating digestive intolerance, and therefore, their administration is intuitively avoided by most physicians in the case of patients with diabetic gastroparesis. However, treatment with long-acting GLP-1 receptor agonist liraglutide was found to have minor or no effects on gastric motility in the recent study on subjects with diabetic neuropathy [84]. Beyond its usual indications in type 2 diabetes therapy, a trial also using liraglutide, this time in type 1 diabetic patients submitted to a hypoglycemic clamp, found no effect of this GLP-1 receptor agonist on gastric emptying or on recovery from hypoglycemia. However, gastric emptying was measured in this study by the less-used method of an absorption test of paracetamol given within a liquid meal [90]. Nevertheless, even if no detrimental effects would occur, choosing short-acting GLP-1 receptor agonist therapies in patients with diabetic gastroparesis seems an illogical and useless option, since their benefits are based on delay of gastric emptying. Patients with type 2 diabetes and slow gastric emptying at baseline may benefit more from treatment with long-acting GLP-1 receptor agonists, while in those with preserved gastric emptying, short-acting GLP-1 receptor agonists may be preferable. Although not yet tested, this hypothesis warrants further investigation.

## 9. Conclusion

Gastric emptying and glycemic control exert an ongoing influence upon each other. Normal rates of gastric emptying, of 1 to 4 kcal/min, correspond to the best balance between intestinal propulsion and absorption of macronutrients (especially carbohydrates), incretin hormone secretion, and postprandial glycemic levels. Contrary to this equilibrium state, higher rates of gastric emptying may induce postprandial hyperglycemia but also represent a compensatory mechanism intervening when hypoglycemia occurs, while slower gastric emptying limits postprandial glycemic excursions or even acts as compensator under hyperglycemic conditions. In diabetic patients, fluctuations in gastric emptying are induced by complex pathophysiological pathways; these fluctuations may have a highly variable, unpredictable time pattern and limited correlations with the severity of clinical manifestations but strongly associate with variations in postprandial glycemic levels. Increased knowledge of this relation between gastric emptying and postprandial glycemic values allowed therapies targeting both factors to be updated, including diets with modified content or incretin-based medications. Future research and development will probably expand the range of both types of interventions, with diets based on reconsidered meal content or sequence and more GLP-1 receptor agonists approved for clinical use.
